# Risk Factors Associated with Stunting among Children Under Five in Timor-Leste

**DOI:** 10.5334/aogh.4199

**Published:** 2023-09-28

**Authors:** Kanae Nomura, Aliza K. C. Bhandari, Emilie Louise Akiko Matsumoto-Takahashi, Osamu Takahashi

**Affiliations:** 1 Graduate School of Public Health, St. Luke’s International University, 3-6-2-5F Tsukiji, Chuo, Tokyo, 104-0045, Japan; 2 Department of Health Policy, National Center for Child Health and Development, 2-10-1 Okura, Setagaya-ku, Tokyo, 157-8535, Japan; 3 Division of Prevention, National Cancer Center, Institute for Cancer Control, 5-1-1 Tsukiji, Chuo-ku, Tokyo, 104-0045, Japan

**Keywords:** stunting, malnutrition, maternal and child health, Timor-Leste, children under five

## Abstract

**Background:**

Undernutrition, including stunting, is the cause of almost 45% of all deaths among children under the age of five. It not only affects child growth but also has a long-term negative influence on cognitive and physical abilities. Timor-Leste has the highest prevalence of child stunting in Southeast Asia. Therefore, this study aimed to identify the prevalence of stunting and factors associated with it.

**Methods:**

This was a cross-sectional study conducted using the Demographic and Health Survey of 2016 for Timor-Leste. The prevalence of stunting among children under five years of age was examined, and bivariable and multivariable logistic regression analysis was conducted to identify the factors associated with stunting.

**Results:**

Among 4,581 children under five years of age, growth in nearly 40% was stunted. The majority of the mothers with stunted children were of age 20–30 years with about 33% having their first baby at ≤19 years of age. Compared to women of <145 cm of height, those of ≥145 cm height had lower likelihood of having a stunted child (OR: 0.62, 95% CI: [0.48–0.80], *p* < 0.001). It was also interesting to note that the risk of stunting was lower among female children than male children [OR: 0.75, 95% CI: (0.64–0.88), *p* < 0.001] in our adjusted model. Similarly, other factors such as wealth index, postnatal care visits, currently breastfeeding, age of the child, and size of the child at birth were also associated with stunting.

**Conclusion:**

The present findings indicate that child stunting in Timor-Leste is mainly associated with maternal and child sociodemographic status. Hence, it is crucial to identify the quality of services provided by health facilities, the involvement of health workers and volunteers, and the intention of mothers to use the health services in Timor-Leste.

## Background

Approximately 720 to 811 million people in the world faced hunger in 2020, with the number of people affected worldwide by hunger increasing in 2020 under the COVID-19 pandemic [1]. The main reasons for hunger are climate change, conflict, and economic stagnation. Shocks from climate change destroy people’s lives, crops, livelihoods, and their ability to be self-sufficient [2]. Conflicts affect food production, which hinders food transportation and prevents food from reaching the hungry. As a result, people become anxious about food security, which causes much trouble, and people may suffer from hunger if food cannot be supplied due to an economic crisis. Thus, food insecurity is also related to malnutrition. The solution to malnutrition is not limited to the individual level but also needs to be addressed at the community, national, and global levels.

According to the World Health Organization (WHO), malnutrition refers to deficiencies or excesses in nutrient intake, an imbalance of vital nutrients, or impaired nutrient use. Stunting, wasting, and underweight are categorized as undernutrition, which is responsible for approximately 45% of child deaths worldwide. These factors are the most common causes of child deaths in low- and middle-income countries. Stunting in children is defined as more than two standard deviations (SD) below WHO’s Child Growth Standards median height-for-age [3]. Stunting increases the risk of death from infections and delays recovery. Also, child stunting is considered to have a long-term negative impact on individuals and society due to reduced cognitive and physical growth, decreased productive capacity and health status, and increased risk of degenerative diseases [4]. From the global perspective, the prevalence of child stunting has diminished steadily for two decades; however, as of 2020, nearly 149.2 million (22.0%) children under the age of five are affected by stunting. As of 2019, more than one in two of all stunted children under five lived in Asia, and two of five lived in Africa. In Southeast Asia, 25.8% of children under five are stunted [5].

Timor-Leste is one of the Southeast Asian countries that gained independence from Indonesia in 2002. People under 20 in Timor-Leste account for over half of its population, making it one of the youngest countries by age of population in Asia. Around one in every five young women marries before age 18, and one in four adolescent women has a child [6].

Although child stunting in Timor-Leste declined from 50.2% to 47.1% between 2013 and 2020, its prevalence in Timor-Leste is still the highest in the Southeast Asian region and the fourth highest in the world [5]. Hence, the prime minister in Timor-Leste launched the large national goal of eliminating hunger and malnutrition in 2021 [7].

Various studies have discussed the relationship between child stunting and risk factors worldwide. Maternal, child, household, and availability of service factors are commonly related to stunting. It is generally known that adolescent pregnant women tend to have a low maternal weight gain, a high risk of anemia, and children with malnutrition. Moreover, there is clear evidence of adolescent social and economic vulnerabilities [8,9]. However, few articles have been published on risk factors of child stunting in Timor-Leste even though these factors exist, and little is known about how maternal characteristics and background contribute to child stunting in Timor-Leste [10]. Not just the sociodemographic status of a mother but also her perinatal status and behavior such as breastfeeding [11] might have a major impact upon the growth and development of a child. Hence, the present study is unique in that it employs such important variables not used in previous studies [10] and examines high stunting among children living in under-studied Timor-Leste.

## Methods

### Study design

This study used data from the 2016 Demographic and Health Survey (DHS) of Timor-Leste obtained from the United States Agency for International Development (USAID). DHS is a nationally representative survey with multi-stage cluster sampling of women of the reproductive age group of 15–49 years. It provides information on such factors as population estimates, health, and nutritional status in more than 90 developing countries worldwide at regular intervals. The first DHS survey of Timor-Leste was carried out in 2009, and the 2016 survey is the latest dataset available to date.

### Study population

The 2016 DHS survey covered a sample of 11,502 households and interviewed 12,607 females aged 15–49 years along with 4,622 males aged 15–59 years. However, we only included women with live births and singleton pregnancy and who had children under five years of age at the time of the survey. We also excluded from this study those who had missing or erroneous records for the height, age, and sex of the child.

### Dependent variable

Stunting was the dependent variable and was defined as the height-for-age of children 0–59 months of age of <–2 SD of the WHO Child Growth Standards median taking into consideration the sex of the child.

### Independent variables

This study included several sociodemographic factors of the mother, institutional factors, behavioral factors, and demographic factors of the child. We included maternal age, age at first birth, educational status, wealth quintiles, place of residence, region (the mother’s municipality of residence), maternal height in centimeters and maternal body mass index along with some pregnancy-related factors such as the number and place of antenatal care (ANC) visits, counseling received on breastfeeding at ANC visits, type of delivery, place of delivery, and postnatal care (PNC) visits. We also included maternal behavioral factors such as early initiation of breastfeeding (if the child was fed breast milk within one hour of birth), infant feeding practices (exclusive breastfeeding if the child was fed only breast milk until six months of life, predominant feeding of the child with plant-based drinks along with breastfeeding, and partial breastfeeding of the child with animal-based drinks or soft solid foods before six months of age) until the child was six months of age, current breastfeeding status, and maternal and child bonding (which was regarded as positive if the mother took the child out, played with them, or read them a book within three days before the survey or if the child was not left alone with other children for more than one hour, and there was child-mother contact immediately after birth).

### Statistical analysis

Univariate analysis was conducted using the chi-square test for categorical variables and Student *t*-test for continuous variables, and weighted frequency and percentage were presented. We also performed bivariable and multivariable logistic regression to identify the factors associated with stunting among the children under five, with careful attention to multicollinearity. A *p* value of <0.05 was considered statistically significant. Statistical analysis was conducted using Stata BE 17.0. We set our survey data first with a stage-level sampling weight variable, primary sampling unit (psu), stratum identifier variable (strata) with Taylor linearized variance estimation before analyzing our dataset and used the “svy” command to address the clustering of the DHS survey data throughout the analysis.

### Ethical approval

This study analyzed the publicly available secondary data of the DHS of Timor-Leste obtained from USAID. The DHS program maintains strict standards for protecting the privacy of respondents and household members in all DHS surveys (https://dhsprogram.com/Methodology/Protecting-the-Privacy-of-DHS-Survey-Respondents.cfm). These surveys have been reviewed and approved by ICF Institutional Review Board (IRB) (ICF IRB FWA00000845). The DHS program is authorized to distribute unrestricted data files for legitimate academic research once researchers register for access to the dataset. We thus obtained permission to access and use the dataset from USAID after it reviewed our proposal.

## Results

Of the 7,206 eligible participants, 4,581 participants had information on age, height, and sex of their child. Of those children, nearly 40% were stunted (*N* = 1,789). The majority of the mothers of those children were of age 20–30 years with about 33% having their first baby at ≤19 years of age. Among woman ≥145 cm in height, 87% of their children were stunted. Similarly, mothers who were normal to underweight had a higher rate of stunting among their children. The rate of stunting was nearly 70% among women without or with less than a secondary education. The richest women had the lowest prevalence of stunting among the children (16.2%). The results also showed that woman residing in rural areas had a rate of stunting among their children nearly three-fourths greater than that of women residing in urban areas. We also found that the rate of stunting was highest among those women residing in the capital city of Timor-Leste, Dili (21.5%), whereas the lowest prevalence was observed in Aileu municipality (3.9%). Among women with <4 ANC visits, 23% of their children were stunted. Similarly, the rate of stunting was slightly more than 50% among women who delivered their baby at home. About 78% of the children of women who did not attend PNC visits were stunted. Similarly, the prevalence of stunting was high among children who were not exclusively breastfed or who lacked positive maternal and child bonding. We also identified a 10% higher rate of stunting among male than female children (Table 1).

**Table 1:** Prevalence of stunting across various sociodemographic characteristics of the study population (*N* = 4,581).


	FREQUENCY (%)		

	NO (*N* = 2,792)	YES (*N* = 1,789)	*P* VALUE

Current maternal age group, years			0.544^1^

≤19	88 (3.3)	49 (2.7)	

20–30	1,424 (51.4)	894 (51.3)	

30–40	999 (35.5)	645 (34.9)	

≥40	281 (9.8)	201 (11.1)	

Maternal age at first birth, years			0.035^1^*

≤19	819 (29.6)	605 (33.0)	

≥20	1,973 (70.4)	1,184 (67.0)	

Maternal height			0.039^1^*

<145 cm	256 (9.4)	221 (12.5)	

≥145 cm	2,510 (89.7)	1,559 (87.0)	

Missing	26 (0.9)	9 (0.5)	

Body mass index			0.904^1^

Underweight	590 (20.5)	363 (19.8)	

Normal weight	1,825 (65.6)	1,194 (66.6)	

Pre-obesity	293 (11.0)	188 (11.0)	

Obesity	56 (1.9)	31 (2.0)	

Missing	28 (1.0)	13 (0.7)	

Education level			<0.001^1^***

No education	632 (22.4)	485 (26.7)	

Less than secondary	1,133 (40.8)	772 (42.9)	

Secondary or above	1,027 (36.9)	532 (30.4)	

Wealth index			0.003^1^**

Poorest	486 (18.1)	373 (20.4)	

Poor	543 (19.1)	382 (21.3)	

Average	559 (19.2)	380 (20.8)	

Rich	635 (20.7)	397 (21.4)	

Richest	569 (23.0)	257 (16.2)	

Area of residence			0.075^1^

Urban	910 (30.8)	492 (26.6)	

Rural	1,882 (69.2)	1,297 (73.4)	

Region			<0.001^1^***

Aileu	206 (3.9)	143 (3.9)	

Ainaro	154 (3.9)	158 (5.9)	

Baucau	187 (9.5)	137 (12.0)	

Bobonaro	207 (7.9)	173 (10.6)	

Covalima	172 (5.9)	135 (6.7)	

Dili	326 (22.7)	184 (21.5)	

Ermera	239 (10.6)	85 (5.4)	

Lautem	209 (5.8)	104 (4.3)	

Liquica	239 (7.0)	151 (6.9)	

Manatuto	219 (4.7)	131 (4.6)	

Manufahi	254 (5.7)	138 (4.8)	

Oecussi	196 (6.9)	115 (6.5)	

Viqueque	184(5.7)	135 (6.9)	

Number of ANC visits			0.126^1^

<4 visits	645 (22.4)	433 (23.0)	

≥4 visits	2,144 (77.5)	1,349 (77.0)	

Missing	3 (<0.001)	7 (0.4)	

Received counseling on breastfeeding at ANC visits			0.542^1^

No	784 (29.6)	534 (30.7)	

Yes	2,006 (70.4)	1,255 (69.3)	

Missing	2 (<0.001)	0 (0.00)	

Type of delivery			0.309^1^

Normal	2,675 (95.9)	1,718 (94.9)	

Cesarean section	116 (4.0)	69 (4.9)	

Missing	1 (<0.001)	2 (0.2)	

Place of delivery			0.068^1^

Home-based delivery	1342 (48.1)	938 (51.6)	

Facility-based delivery	1450 (51.9)	851 (48.4)	

Missing	20 (0.7)	16 (0.9)	

PNC check of baby within two months of life			0.878^1^

No	2,160 (77.8)	1,389 (78.3)	

Yes	598 (21.0)	379 (20.4)	

Missing	34 (1.2)	21 (1.3)	

Early initiation of breastfeeding			0.303^1^

Within one hour of birth	2,135 (76.4)	1,337 (74.1)	

After one hour of birth	575 (20.7)	396 (22.9)	

Missing	82 (2.9)	56 (3.1)	

Infant feeding practice within first six months of life			0.226^1^

Exclusive breastfeeding	14 (0.6)	10 (0.4)	

Predominant feeding	1,574 (55.1)	1,033 (57.9)	

Partially breastfeeding	1,120 (41.2)	682 (37.9)	

Missing	84 (3.1)	64 (3.7)	

Currently breastfeeding			<0.001^1^***

No	1,293 (47.3)	1,036 (59.9)	

Yes	1,499 (52.7)	753 (40.1)	

Having positive maternal and child bonding			0.173^1^

Negative	1,376 (49.6)	955 (53.3)	

Positive	1,408 (50.2)	828 (46.4)	

Missing	8 (2.7)	6 (2.9)	

Child’s age in months, mean (SD)	20.49 (16.5)	25.84 (14.9)	<0.001^2^***

Child’s sex			<0.001^1^***

Male	1,396 (49.0)	986 (55.0)	

Female	1,396 (51.0)	803 (45.1)	

Child’s size at birth			0.061^1^

Very large	278 (9.9)	136 (7.6)	

Larger than average	252 (8.5)	163 (8.8)	

Average	1,628 (57.0)	1,031 (56.0)	

Smaller than average	53 (2.0)	40 (3.0)	

Very small	119 (4.2)	97 (5.6)	

Missing	462 (18.4)	322 (19.2)	

Birth order			0.064^1^

First	616 (22.4)	324 (18.9)	

Second	549 (19.7)	356 (21.1)	

Third	450 (16.1)	314 (17.7)	

Fourth or more	1,177 (41.9)	795 (42.3)	


*N* = number of participants, ANC = antenatal care, PNC = postnatal care, *SD* = standard deviation. ^1^Chi-square test, ^2^Student *t*-test. * *p* < 0.05, ** *p* < 0.01, *** *p* < 0.001.

The results of the bivariable and multivariable logistic regression analysis are shown in Table 2. Our bivariable logistic regression analysis revealed several factors that were significantly associated with stunting among children, such as maternal age at first birth, maternal height, educational level, wealth index, municipality of residence, current breastfeeding status, child’s age, child’s sex, child’s size at birth, and birth order. However, after adjusting for the covariates under analysis, we found maternal height, wealth index, region (municipality of residence), PNC check of baby within two months of life (postnatal visit), currently breastfeeding, child’s age in months, child’s sex, and child’s size at birth to be the independent predictors of stunting among the children under five years of age.

**Table 2:** Bivariable and multivariable logistic regression analysis (*N* = 3,553).


	COR (95% CI)	AOR (95% CI)

Current maternal age group, years		

≤19	1	1

20–30	1.20 (0.80–1.80)	1.05 (0.66–1.67)

30–40	1.18 (0.78–1.77)	0.84 (0.47–1.49)

≥40	1.35 (0.87–2.09)	0.76 (0.40–1.43)

Maternal age at first birth, years		

≤19	1	1

≥20	0.85 (0.74–0.99) *	0.92 (0.75–1.11)

Maternal height		

<145 cm	1	1

≥145 cm	0.73 (0.59–0.90) **	0.62 (0.48–0.80) ***

Body mass index		

Underweight	1	1

Normal weight	1.06 (0.87–1.29)	1.12 (0.89–1.41)

Pre-obesity	1.05 (0.78–1.41)	0.96 (0.68–1.36)

Obesity	1.09 (0.64–1.85)	1.03 (0.55–1.95)

Education level		

No education	1	1

Less than secondary	0.88 (0.74–1.05)	0.85 (0.69–1.05)

Secondary or above	0.69 (0.56–0.85) **	0.81 (0.60–1.08)

Wealth index		

Richest	1	1

Poorest	1.60 (1.19–2.15) **	1.61 (1.13–2.28) **

Poor	1.59 (1.20–2.10) **	1.65 (1.04–2.31) **

Average	1.54 (1.12–2.13) **	1.41 (0.99–2.00)

Rich	1.47 (1.16–1.86) **	1.31 (1.01–1.71) *

Area of residence		

Urban	1	1

Rural	1.23 (0.98–1.54)	1.13 (0.90–1.42)

Region		

Dili	1	1

Aileu	1.06 (0.73–1.54)	0.82 (0.57–1.19)

Ainaro	1.59 (1.10–2.29) *	1.17 (0.79–1.75)

Baucau	1.33 (0.94–1.88)	1.08 (0.77–1.51)

Bobonaro	1.41 (1.00–1.98)	0.90 (0.65–1.25)

Covalima	1.18 (0.83–1.70)	0.74 (0.47–1.16)

Ermera	0.54 (0.35–0.84) **	0.32 (0.20–0.52) ***

Lautem	0.78 (0.56–1.10)	0.63 (0.44–0.91) *

Liquica	1.04 (0.73–1.49)	0.74 (0.51–1.06)

Manatuto	1.03 (0.75–1.43)	0.94 (0.64–1.37)

Manufahi	0.89 (0.63–1.26)	0.55 (0.38–0.78) **

Oecussi	1.00 (0.68–1.47)	0.58 (0.39–0.86) **

Viqueque	1.28 (0.88–1.87)	0.82 (0.55–1.21)

Number of ANC visits		

<4 visits	1	1

≥4 visits	0.97 (0.81–1.16)	0.97 (0.77–1.22)

Received counseling on breastfeeding at ANC visits		

No	1	1

Yes	0.95 (0.80–1.13)	0.99 (0.80–1.23)

Place of delivery		

Home-based delivery	1	1

Facility-based delivery	0.87 (0.74–1.01)	0.94 (0.74–1.18)

PNC check of baby within two months of life		

No	1	1

Yes	0.96 (0.81–1.15)	0.80 (0.65–0.99) *

Time from birth to first breastfeeding		

Within one hour of birth	1	1

After one hour of birth	1.14 (0.96–1.35)	1.16 (0.95–1.41)

Exclusive breastfeeding		

No	1	1

Yes	0.73 (0.29–1.79)	0.61 (0.23–1.65)

Currently breastfeeding		

No	1	1

Yes	0.60 (0.52–0.70) ***	0.77 (0.62–0.96) *

Having positive maternal and child bonding		

Negative	1	1

Positive	0.86 (0.72–1.03)	0.95 (0.77–1.18)

Child’s age in months	1.02 (1.02–1.03) ***	1.02 (1.01–1.03) ***

Child’s sex		

Male	1	1

Female	0.79 (0.69–0.90) ***	0.75 (0.64–0.88) ***

Child’s size at birth		

Very large	1	1

Larger than average	1.36 (1.00–1.85)	1.37 (0.95–1.98)

Average	1.28 (0.98–1.68)	1.31 (0.96–1.78)

Smaller than average	1.89 (1.12–3.21) *	1.89 (1.08–3.34) *

Very small	1.73 (1.20–2.50) **	1.45 (0.97–2.17)

Birth order		

First	1	1

Second	1.27 (1.02–1.58) *	1.28 (0.96–1.66)

Third	1.30 (1.06–1.60) *	1.29 (1.00–1.66)

Fourth or more	1.20 (1.00–1.43) *	1.22 (0.90–1.66)


*N* = number of participants, cOR = crude odds ratio, aOR = adjusted odds ratio, CI = confidence interval, ANC = antenatal care, PNC = postnatal care. * *p* < 0.05, ** *p* < 0.01, *** *p* < 0.001.

In detail, compared to women of <145 cm in height, those ≥145 cm in height had a 38% lower likelihood of having a stunted child (OR: 0.62, 95% CI: [0.48–0.80], p < 0.001). We also found that the poorest women and poor women were, respectively, 1.61 (95% CI: [1.13–2.28], *p* value < 0.01) and 1.65 (95% CI: [1.04–2.31], *p* < 0.01) times more likely to have stunted children compared to the richest women after adjusting for all covariates under analysis. Additionally, women residing in Ermera municipality (OR: 0.32, 95% CI: [0.20–0.52], *p* < 0.001), Lautem municipality (OR: 0.63, 95% CI: [0.44–0.91], *p* < 0.05), Manufahi municipality (OR: 0.55, 95% CI: [0.38–0.78], *p* < 0.01), and Oecussi municipality (OR: 0.58, 95% CI: [0.39–0.86], *p* < 0.01) had a lower likelihood of having stunted children than those residing in Dili municipality, the capital of Timor-Leste, after adjusting for the covariates.

We also found that currently breastfeeding, child’s age in months, child’s sex, and child’s size at birth to be the independent predictors of stunting among the children under five years of age. For example, women who were currently breastfeeding had a 23% lower risk of stunting among their children compared to their counterparts (OR: 0.77, 95% CI: [0.62–0.96], *p* < 0.05) after controlling for confounders. It was also interesting to note that the risk of stunting was 25% lower among the female children than male children (OR: 0.75, 95% CI: [0.64–0.88], *p* < 0.001) in our adjusted model. Compared to very large children, those who were born smaller than average size had a significantly higher likelihood of stunting (OR: 1.89, 95% CI: [1.08–3.34], *p* < 0.05) after controlling for all other covariates under analysis (Table 2).

## Discussion

The present study examined the prevalence of stunting in Timor-Leste and identified the factors associated with it. Several factors such as maternal height, wealth index, region (municipality of residence), PNC check of baby within two months of life (postnatal visit), currently breastfeeding, child’s age in months, child’s sex, and child’s size at birth were significantly associated with stunting among children under five years of age.

First, maternal height was significantly associated with stunting among children. Many researchers in the past have pointed out that maternal height is highly associated with child stunting [12,13]. The fact is that maternal short stature affects the growth of children, which means this condition can be inherited from the mother. In fact, some researchers showed that Timor-Leste was one of the countries with the shortest height for both men and women [14]. The possibility of long-term malnutrition from child to adult in Timor-Leste again suggests the need for intervention.

Second, wealth index had an association with child stunting. As many previous studies have shown, the economic condition of the household also affects a child’s health. Our results showed that the extent of economic conditions increases the risk of child stunting in Timor-Leste. This may suggest that household wealth determines the choice of food, health service, or acknowledgment of a child’s health. In Timor-Leste, more than two-thirds of the population live in rural areas, depending on agriculture for their livelihood. The national survey of the World Bank estimated that around 42% of people living in Timor-Leste lived below the poverty line [15].

Third, we also found regional differences in child stunting. Our findings indicated that compared to women in the capital of Timor-Leste, those from Ermera, Lautem, Manufahi, and Oecussi have a lower risk of stunting. This may suggest that the environment in which the mother lives can have a great impact on her child’s growth and development. Similarly, the availability of resources such as health care facilities, health care professionals, and accessibility to health facilities might have played a vital role in bringing these disparities in stunting prevalence across various municipalities of Timor-Leste. Equitable access to health services, especially for pregnant women and postnatal women, should be emphasized to reduce this disparity. On the other hand, according to the result of the integrated food security phase classification (IPC) analysis report on the chronic food insecurity situation in Timor-Leste in 2018 [16], the IPC level of the municipalities of Ermera, Manufahi, and Oecussi yielded severe chronic food insecurity. Thus, the current results showed a negative association between child stunting and food insecurity. This might be due to the sampling of the DHS data. The data we used may have been taken in areas where food is relatively accessible. Therefore, further data analysis is required.

Fourth, mothers attending PNC visits within two months postpartum significantly reduce the risk of child stunting. Health service utilization factors such as ANC visits, frequency, and quality of service provided, place of delivery, and PNC visits might have a positive impact on the prevention of stunting [17]. Health professionals or community health care volunteers can educate pregnant and breastfeeding women regarding the health of the child and mother through ANC and PNC visits. Similarly, growth monitoring of the child after birth, which is usually performed during postnatal visits in Timor-Leste, is important to detect malnutrition such as stunting. The health care centers provide nutritional supplements for the children who have growth deficits according to the government policy, and this has a huge impact in reducing stunting and other forms of malnutrition in Timor-Leste.

Moreover, child’s sex and age were significantly associated with child stunting. Excess male morbidity and mortality are almost universally reported and widely acknowledged in neonatal medicine and infant health communities [18]. In line with this, we found significant differences in the prevalence of stunting in the male children compared to their female counterparts, which is consistent with previous findings [19]. According to other previous studies, stunting was statistically associated with increasing child age in children aged 0–59 months. Our results confirmed this finding [20].

Infant and young child feeding practices during the first two years of life play a crucial role in a child’s growth and development [21]. A previous study revealed that initiating breastfeeding promptly, providing colostrum, and appropriate weaning are essential for preventing undernutrition among children under five [22]. In our study, only currently breastfeeding was associated with child stunting. It is possible that there is a deficiency in the quantity or quality of breast milk due to maternal malnutrition and that breastfeeding is not the primary but a supplementary practice in Timor-Leste.

Finally, the child’s size at birth was associated but birth order was not. Small birth size may indicate poor maternal nutrition, preterm birth, or disease during pregnancy. Therefore, intervention before childbirth might be a practical measure to reduce the incidence of child stunting. Although birth order and child stunting correlate with each other in some studies, our results showed that birth order was not significantly associated with stunting among children [17]. One possible reason for birth order not always being associated with the risk of stunting is that not only birth order but also the interval between births is related to malnutrition. One study showed that children born following a long birth interval have a lower risk of malnutrition for all birth orders. The importance of birth intervals decreases with increasing birth order [23]. Thus, future studies may need to assess the relationship between birth order and birth intervals.

### Strengths and limitations

Although Timor-Leste is poorly studied and has high rate of stunting, the present study provided evidence on the association between stunting and maternal sociodemographic and pregnancy-related factors. The study used a nationally representative sample, and hence, the results could be generalized to the entire population of Timor-Leste. However, this study has a few limitations. First, there were many missing measurement values for the height of the children, which was a key outcome variable of this study. Second, the maternal and child health situation in Timor-Leste might have changed after the COVID-19 pandemic. Although this study used the latest available DHS dataset, that from the year 2016, the results might not portray the current situation in terms of the prevalence of stunting in Timor-Leste.

## Conclusions

This study identified a high prevalence of stunting in Timor-Leste. Overall, child stunting in Timor-Leste was mostly associated with maternal and child sociodemographic factors and the quality and availability of health care services. There is a disparity across various wealth quintiles and regions of Timor-Leste, suggesting that there are certain groups at high risk for stunting. Hence, it is crucial to identify the quality of services provided by health facilities, the involvement of health workers and volunteers in improving the maternal and child health outcomes, and the intention of mothers to use the health services in Timor-Leste. Promoting maternal behavior such as breastfeeding practice in PNC might be especially effective for child stunting in Timor-Leste. Furthermore, ensuring nutritional food intake and improving the accessibility to food for pregnant women is urgently required in Timor-Leste.

## Data Accessibility Statement

The datasets used and/or analyzed during the current study were obtained from the DHS program, which is a publicly available database.

## Figures and Tables

**Table:** 

ANC	Antenatal care
CI	Confidence interval
DHS	Demographic and Health Survey
IPC	Integrated food security phase classification
PNC	Postnatal care
*SD*	Standard deviation
UNFPA	United Nations Population Fund
UNICEF	United Nations Children’s Fund
USAID	United States Agency for International Development
WHO	World Health Organization
aOR	Adjusted odds ratio
cOR	Crude odds ratio
